# Simultaneous Detection and Differentiation of SARS-CoV-2, Influenza A/B, and Respiratory Syncytial Viruses in Respiratory Specimens Using the VitaSIRO *solo*™ SARS-CoV-2/Flu/RSV Assay

**DOI:** 10.3390/diagnostics15172249

**Published:** 2025-09-05

**Authors:** Ralph-Sydney Mboumba Bouassa, Sarah Lukumbisa, Laurent Bélec

**Affiliations:** 1 Ecole Doctorale Régionale (EDR) d’Afrique Centrale en Infectiologie Tropicale, Franceville BP 876, Gabon; ralphsmbouassa@montfort.on.ca; 2 Institut du Savoir Montfort, Montfort Hospital, Ottawa, ON K1K 0T2, Canada; 3 Department of Family Medicine, Faculty of Medicine, University of Ottawa, Ottawa, ON K1N 6S1, Canada; 4 Licence Sciences de la Vie et de la Terre, Parcours Biologie-Santé, Faculté des Sciences et Technologie, Université Paris-Est Créteil (UPEC), 94000 Créteil, France; sarah.lukumbisa@etu.u-pec.fr; 5 Laboratory of Virology, Hôpital Européen Georges Pompidou, Assistance Publique-Hôpitaux de Paris (AP-HP), 75015 Paris, France; 6 Faculté de Médecine Paris Descartes, Université Paris Cité, 75006 Paris, France

**Keywords:** SARS-CoV-2, influenza A virus, influenza B virus, respiratory syncytial virus, triplepidemic, multiplex, PCR, point-of-care, 2024–2025 autumn-winter season, France

## Abstract

**Background/Objectives:** The concurrent circulation of SARS-CoV-2 with influenza A and B viruses and respiratory syncytial virus (RSV) represents a new diagnostic challenge in the post-COVID-19 area, especially considering that these infections have overlapping clinical presentations but different approaches to treatment and management. Multiplexed molecular testing on point-of-care platforms that focus on the simultaneous detection of multiple respiratory viruses in a single tube constitutes a useful approach for diagnosis of respiratory infections in decentralized clinical settings. This study evaluated the analytical performances of the VitaSIRO *solo*™ SARS-CoV-2/Flu/RSV Assay performed on the VitaSIRO *solo*™ Instrument (Credo Diagnostics Biomedical Pte. Ltd., Singapore, Republic of Singapore). **Methods:** With a view to accreditation, the criteria of the 2022-revised EN ISO 15189:2022 norma were applied for the retrospective on-site verification of method using anonymized respiratory specimens collected during the last 2024–2025 autumn–winter season in France. **Results:** Usability and satisfaction were comparable to current reference point-of-care platforms, such as the Cepheid GeneXpert^®^ Xpress System (Cepheid Diagnostics, Sunnyvale, CA, USA). Repeatability and reproducibility (2.34–4.49% and 2.78–5.71%, respectively) demonstrated a high level of precision. The platform exhibited a low invalid rate (2.9%), with most resolving on retesting. Analytical performance on 301 clinical samples showed high overall sensitivities: 94.8% for SARS-CoV-2 (Ct ≤ 33), 95.8% for influenza A and B viruses, 95.2% for RSV, and 95.4% for all viruses. Specificities were consistently high (99.2–100.0%). False negatives (2.6%) were predominantly associated with high Ct values. Agreement with the comparator reference NeuMoDx™ Flu A-B/RSV/SARS-CoV-2 Vantage Assay (Qiagen GmbH, Hilden, Germany) was almost perfect (Cohen’s κ 0.939–0.974), and a total of 91.1%, 94.8%, and 100.0% of Ct values were within the 95% limits of agreement for the detection of SARS-CoV-2, influenza A and B viruses, and RSV, respectively, by Bland–Altman analyses. Passing–Bablok regression analyses demonstrated good Ct values correlation between VitaSIRO *solo*™ and NeuMoDx™ assays, with a slight, non-significant, positive bias for the VitaSIRO *solo*™ assay (mean absolute bias +0.509 to +0.898). **Conclusions:** These findings support VitaSIRO *solo*™ Instrument as a user-friendly and reliable point-of-care platform for the rapid detection and differentiation of SARS-CoV-2, influenza A and B viruses, and RSV responding to the EN ISO 15189:2022 criteria for accreditation to be implemented in hospital or decentralized settings.

## 1. Introduction

The emergence of the coronavirus disease 2019 (COVID-19) caused by severe acute respiratory syndrome coronavirus 2 (SARS-CoV-2) has resulted in an unprecedented global pandemic leading to significant morbidity and mortality, particularly among older and vulnerable adult populations [1,2]. During the pandemic’s early stages, intensified public health measures were implemented worldwide to mitigate disease spread, including travel restrictions, lockdowns, messaging on handwashing, use of face coverings, and physical distancing. As the pandemic progressed, exceptional decreases in the circulation of traditional seasonal respiratory viruses, including influenza A and B viruses and respiratory syncytial virus (RSV), were unexpectedly increasingly reported [3,4,5]. However, more than 5 years after the beginning of the COVID-19 epidemic, the restrictions to prevent community-acquired respiratory infections have once again all but disappeared from social life. Therefore, the cocirculation of SARS-CoV-2 with influenza A and B viruses and RSV has progressively emerged, often occurring outside their traditional seasonal patterns [6,7,8,9,10,11,12,13,14,15,16,17,18,19,20,21,22].

Patients infected with SARS-CoV-2, influenza A or B viruses, and RSV have overlapping clinical manifestations, with very similar symptoms complicating the reliance on clinical diagnosis alone for their differentiation [23,24]. Otherwise, patients infected with these viruses have quite different approaches to treatment and clinical management. Unlike common-cold viruses, infection with these four viruses is often associated with fever and other systemic manifestations that may be coupled with severe outcomes, especially in the elderly [25,26]. Independently of SARS-CoV-2 infection, influenza viruses and RSV have contributed to severe respiratory infections that are a significant cause of morbidity, mortality, and hospital admissions each year [27]. Possible co-infection with SARS-CoV-2 and other concurrent respiratory viruses, with influenza and RSV being the commonest, could also have important implications for clinical management and may increase the disease severity of COVID-19 patients [28,29]. Otherwise, SARS-CoV-2 and influenza viruses, in particular, have a significant impact on transmission inside and outside hospitals. Therefore, rapid diagnosis combined with infection prevention measures is of paramount importance to stop the chain of transmission [30,31]. In other respects, without a definitive diagnosis, patients with viral respiratory infections are more likely to receive unnecessary antibiotic treatment. Finally, rapid diagnosis of SARS-CoV-2, influenza A and B viruses, and RSV, in conjunction with infection prevention measures, is essential for patient management and infection control, as well as public health surveillance and response.

The co-occurrence of SARS-CoV-2 with other respiratory viral infections, and possible codetection, poses a new diagnostic challenge for clinical microbiology laboratories. The primary method for diagnosing SARS-CoV-2, influenza viruses, and RSV infection was singleplex real-time reverse transcription-polymerase chain reaction (rRT-PCR) [32]. Unfortunately, conducting singleplex rRT-PCR for the four viruses can be labor-intensive and time-consuming, resulting in delayed patient management. An optimal diagnostic algorithm for testing patients with an influenza-like disease is likely a multiplex assay that combines the four targets to simultaneously test for SARS-CoV-2, influenza A virus, influenza B virus, and RSV. Multiplex rRT-PCR, which enables the simultaneous detection of multiple respiratory viruses in a single tube, provides an ideal solution to this issue [33,34,35,36]. Many multiplex methods are currently available for the simultaneous detection of a variety of respiratory viruses with various throughput capacity, turn-around times, and/or cost [10,37,38,39,40,41,42,43,44,45,46,47,48,49,50,51,52,53,54,55,56,57,58,59,60,61]. Some molecular assays must be run in centralized, certified laboratories by trained technologists, while others can be performed by staff with minimal laboratory training at point-of-care or near-patient. In particular, multiplex point-of-care tests, allowing detection and differentiation of the most common respiratory viruses, including SARS-CoV-2, influenza A and B viruses, and RSV, are particularly interesting as powerful decision-making tools for patient management in a decentralized diagnostic approach, especially in emergency departments [46,48,59].

In search for a platform which can be performed at point-of-care in the emergency departments of our institution (AP-HP), we had previously evaluated during the first year of the COVID-19 epidemic in Paris the VitaPCR™ SARS-CoV-2 Assay and the VitaPCR™ Flu A&B Assay performed on the first generation VitaPCR™ Instrument developed by Credo Diagnostics Biomedical Pte. Ltd. (Singapore, Republic of Singapore) [62,63]. In the present study, we retrospectively evaluated, during the last 2024–2025 autumn–winter season in France, the rRT-PCR VitaSIRO *solo*™ SARS-CoV-2/Flu/RSV Assay (Credo Diagnostics Biomedical Pte. Ltd.) performed on the novel second-generation fully automated point-of-care VitaSIRO *solo*™ Instrument (Credo Diagnostics Biomedical Pte. Ltd.) for the simultaneous detection and differentiation of SARS-CoV-2, influenza A and B viruses, and RSV. With accreditation in mind, we applied the on-site verification of method with criteria of the 2022-revised EN ISO 15189:2022 norma, the latest revision of the international standard for medical laboratories, which now includes significant updates that address point-of-care testing [64,65].

## 2. Materials and Methods

### 2.1. Clinical Specimens

This monocenter, retrospective study included nasopharyngeal specimens from adult patients (≥18 years old) suffering from influenza-like signs and symptoms for 35 weeks, from 16 September 2024 [week 38 (W38)] to 18 May 2025 (W20). Nasopharyngeal specimens were collected using nasopharyngeal nylon-flocked swabs with breakpoint at 80 mm (δswab^®^, Deltalab, Rubí Barcelona, Spain; catalog reference: 304305KF) collected by a nurse or physician using standardized methods. After sampling, the nasopharyngeal swab was discharged into a vial containing 3 mL of universal virus transport medium (Deltalab). Remnant nasopharyngeal swab specimens that were destined to be thrown away were collected after the standard care diagnostic testing was performed and kept frozen at −30 °C until use. The respiratory specimens were completely anonymized, with no knowledge of the patient’s age, sex, or hospitalization or consultation department. The only information retained was the number of the week during which the sample was collected, and the qualitative and possibly semi-quantitative [cycle threshold (Ct) in arbitrary unit (a.u.)] results obtained by the reference assays used in the laboratory.

### 2.2. Reference Molecular Testing for Respiratory Viruses

The NeuMoDx™ Flu A-B/RSV/SARS-CoV-2 Vantage Assay (Qiagen GmbH, Hilden, Germany) performed on the NeuMoDx™ 288 Molecular System, a random access, high-throughput platform, was used as the principal reference comparator assay for respiratory viruses. The assay is a multiplex, rapid, and automated qualitative in vitro rRT-PCR diagnostic test intended for simultaneous direct detection and differentiation of SARS-CoV-2, influenza A virus, influenza B virus, and RSV from nasopharyngeal swab samples in transport medium. The assay combines automated RNA extraction and amplification/detection of viruses by rRT-PCR. The NeuMoDx™ Flu A-B/RSV/SARS-CoV-2 Vantage Assay targets the conserved region of SARS-CoV-2 Nsp2 gene and regions in the M genes of influenza A virus, influenza B virus, and RSV A and B genomes. Individual Ct values are recorded for each target gene. The NeuMoDx™ Flu A-B/RSV/SARS-CoV-2 Vantage Assay is a reliable assay for syndromic testing of SARS-CoV-2, influenza A and B viruses, and RSV, according to previous reported evaluations [49,51,55].

The FilmArray Respiratory Panel 2.1 (Bio-Fire Diagnostics, Salt Lake City, UT, USA; catalog reference: 423742) was used as an alternative reference comparator assay for respiratory viruses, occasionally prescribed at the request of clinicians. The assay is a cartridge-based multiplex nested rRT-PCR test that utilizes melting curve analysis for the simultaneous detection of 22 different respiratory pathogens, including SARS-CoV-2, influenza A virus, influenza B virus, and RSV A and B, in a single nasopharyngeal sample with high sensitivity and specificity [37,38,39,41]. The test was carried out on the BioFire^®^ FilmArray^®^ Torch System (Bio-Fire Diagnostics), including a panel-specific software module, and accompanying system-specific software. No Ct of target genes can be provided.

Outside of epidemic periods of influenza infections and/or RSV infection, the CE-IVD Xpert^®^ Xpress CoV-2 plus test (Cepheid Diagnostics, Sunnyvale, CA, USA) performed on the Cepheid GeneXpert^®^ Xpress System (Cepheid Diagnostics) was used for SARS-CoV-2 detection and serves as a reference comparator fully validated assay [42,43,50]. No Ct of target genes can be shown.

The virology laboratory at Hôpital Européen Georges Pompidou is otherwise accredited according to EN ISO 15189:2022 for the scope “VIROH,” which includes specialized virology markers.

### 2.3. Simultaneous Detection of SARS-CoV-2, Influenza A Virus, Influenza B Virus, and RSV by VitaSIRO solo™ SARS-CoV-2/Flu/RSV Assay

**Figure 1:**
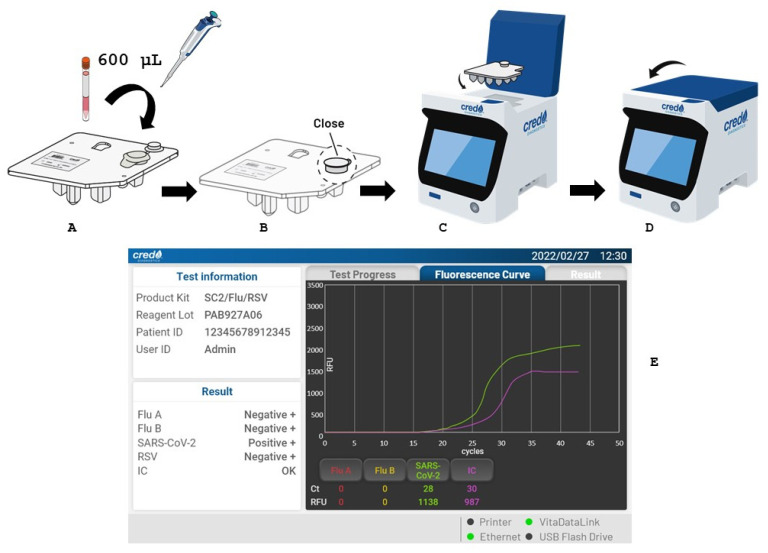
Preanalytical and analytical workflow for respiratory virus detection using the VitaSIRO *solo*™ Instrument (Credo Diagnostics Biomedical Pte. Ltd.): (**A**). A nasal or nasopharyngeal specimen is collected using a nylon-flocked swab and placed into a 3 mL plastic tube containing universal transport medium. The tube is then inverted 5–10 times with the cap tightly secured to mix the sample; (**B**). After removing the foil from the sample well, 600 µL of the specimen is transferred into the sample chamber of the VitaSIRO *solo*™ SARS-CoV-2/Flu/RSV Assay cartridge; (**C**). The cartridge lid is closed, and the cartridge is inserted into the VitaSIRO *solo*™ Instrument; (**D**)**.** After closing the instrument lid, the analysis runs for approximately 40 min; (**E**). View of the real-time PCR screen during the analysis. The standalone VitaSIRO *solo*™ Instrument utilizes a patented microfluidic cartridge that integrates the entire molecular diagnostic process—from sample preparation to result—encompassing nucleic acid extraction, real-time PCR amplification, and amplicon detection. The cartridge features an “all-in-one” sealed design with all necessary reagents pre-loaded. The instrument supports multiplex detection of up to 6 targets and accommodates a broad range of sample types. Fluorescence curves can be monitored live, and a “beep” indicates completion. Final results are automatically displayed on the screen.

The VitaSIRO *solo*™ SARS-CoV-2/Flu/RSV Assay (Credo Diagnostics Biomedical Pte. Ltd.) is a fully automated multiplex molecular in vitro diagnostic test performed on the VitaSIRO *solo*™ Instrument (Figure 1; https://www.credodxbiomed.com/fr/our-products/vitasiro-sol/, accessed on 7 August 2025). The assay enables the simultaneous qualitative detection and differentiation of SARS-CoV-2, influenza A, influenza B, and RSV RNA from nasal or nasopharyngeal swab specimens collected from individuals presenting with symptoms of respiratory infection.

Designed for point-of-care use, VitaSIRO *solo*™ platform integrates the entire molecular diagnostic workflow (nucleic acid extraction, purification, amplification, and detection) within a sealed, single-use, microfluidic cartridge. The acronym “SIRO” means “Sample-In-Result-Out”, suggesting simplicity, automation, and user-centric design, and highlighting the minimal user intervention required. Indeed, in a point-of-care setting, ease of use is paramount. “SIRO” implies that the user simply introduces the sample, and the device handles the complex molecular processes internally, ultimately presenting a clear result.

The VitaSIRO *solo*™ Instrument constitutes an automated analyzer integrating a full workflow of nucleic acid extraction and rRT-PCR technology in a microfluidic cartridge-based design. This standalone instrument is portable. It features a 7 inch color touch screen with user-friendly interface that allows intuitive display and a barcode scanner. The instrument’s computing is compatible with most laboratory informatic systems. The VitaDataLink™ Connectivity solution via HL7 message enables connection of up to 8 instruments with centralized data management. The VitaSIRO *solo*™ Instrument receives the loaded cartridge. It has a micro-pump that applies a controlled pressure to circulate liquids within the cartridge, according to a patented liquid transfer technology. The instrument precisely rotates the cartridge’s central valve to establish the fluidic connections between chambers according to the current stage of analysis. The instrument also controls the temperature cycles within the cartridge, with rapid heating and cooling, allowing accurate temperature cycling. As the PCR reaction progresses, the instrument’s optical system monitors the fluorescence signals in real-time. The increase in fluorescence directly correlates with the amount of amplified DNA. The instrument software analyzes the real-time fluorescence curves to determine the Ct value for each target. The operating environment temperature ranges from +10 °C to +38 °C. Training to use this analyzer is minimal, lasting about 1 h for a laboratory technician.

The all-in-one sealed cartridge of the VitaSIRO *solo*™ SARS-CoV-2/Flu/RSV Assay contains the sample chamber, miniaturized channels, and 13 reaction chambers containing all the pre-loaded reagents and components necessary for nucleic acid extraction, comprising the steps of sample lysis, magnetic beads binding, washing (to remove impurities and PCR inhibitors) and final nucleic acid elution, and rRT-PCR amplification with all necessary reagents (primers, probes, DNA polymerase, dNTPs). At each stage of the analysis, the reaction fluid moves under the controlled pressure from one reaction chamber to another, thanks to the microchannels that are brought into contiguity by the cartridge’s central valve. The cartridge can be stored at room temperature (+4–30 °C).

To perform the test, 600 µL of the specimen—collected using sterile nylon-flocked swabs and placed in either manufacturer-supplied buffer or universal viral transport medium—is transferred directly into the sample chamber of the cartridge, which is then loaded into the instrument (Figure 1). The assay employs primer/probe sets targeting the nucleocapsid (N) gene of SARS-CoV-2, the matrix (M) gene of influenza A, the non-structural (NS) gene of influenza B, the nucleocapsid (N) gene of RSV A and B, and an in vitro synthesized and encapsulated RNA used as an internal control meant to monitor sample processing and the potential presence of PCR inhibitors.

**Figure 2:**
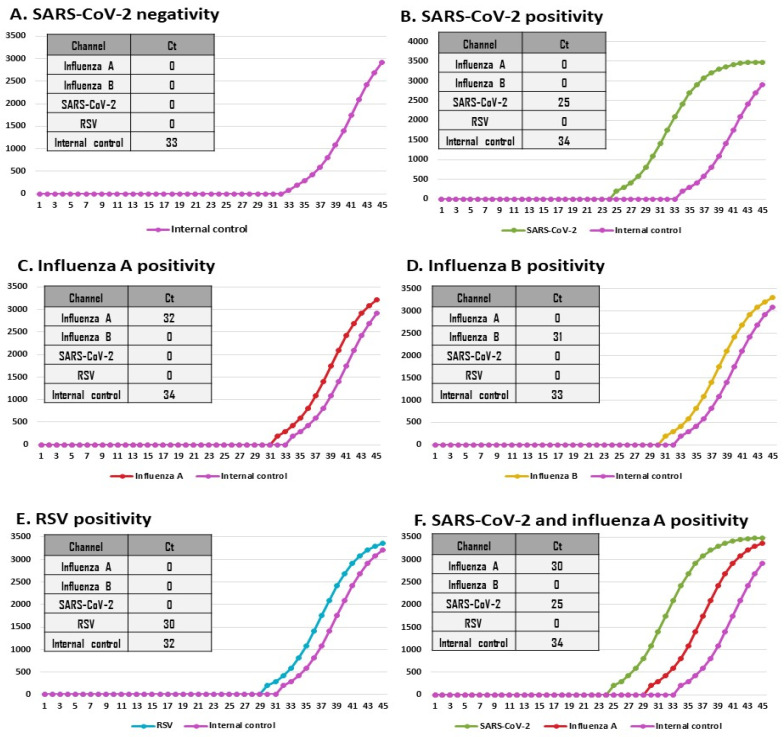
The VitaSIRO *solo*™ SARS-CoV-2/Flu/RSV Assay (Credo Diagnostics Biomedical Pte. Ltd.) targets the N gene of SARS-CoV-2, the M gene of influenza A, the NS gene of influenza B, the N gene of RSV A and B, and an internal control, providing individual Ct values for each target gene. By clicking on the “Fluorescence Curve” tab, the fluorescence curve plot is displayed on the screen of the VitaSIRO *solo*™ Instrument (Credo Diagnostics Biomedical Pte. Ltd.). The VitaSIRO *solo*™ SARS-CoV-2/Flu/RSV Assay enables the identification of five types of amplification curve profiles, including negative test result with only the purple amplification curve corresponding to the internal RNA control, positive test result for SARS-CoV-2 with green amplification curve of the N gene, positive test for influenza A with a dark red amplification curve of M gene, positive test for influenza B with an orange amplification curve of NS gene, and positive test for RSV with a blue amplification curve of N gene, as shown in following representative fluorescence amplification curves and corresponding Ct values: (**A**). Negative result for SARS-CoV-2; (**B**). Positive result for SARS-CoV-2; (**C**). Positive result for influenza A; (**D**). Positive result for influenza B; (**E**). Positive result for RSV; (**F**). Example of co-infection with SARS-CoV-2 and influenza A. For each panel, the amplification curve is displayed alongside a table summarizing the corresponding Ct values for the detection channels: SARS-CoV-2, influenza A (Flu A), influenza B (Flu B), RSV, and internal control. A Ct value of “0” indicates no amplification (target not detected). The *x*-axis represents the number of cycles; the *y*-axis represents the fluorescence intensity in arbitrary units. Ct: Cycle threshold; RSV: Respiratory syncytial virus.

Detection is achieved through real-time fluorescence monitoring across five independent optical channels (fluorescence detection between 450 and 750 nm), allowing for the visualization of amplification fluorescence curves and the reporting of Ct values for each target gene in real time (Figure 2). The total time to result is approximately 40 min. All procedures were conducted following the manufacturer’s instructions for use. After the PCR run, the instrument automatically analyzes the data and reports the results (positive/negative for each target) on its touchscreen interface. It can also transfer these results to laboratory information systems via various protocols.

The hospital quality control (QC) test was carried once a week using positive and negative external controls supplied by the manufacturer. The positive control (VitaSIRO *solo*™ SARS-CoV-2/Flu/RSV External control set, Credo Diagnostics Biomedical Pte. Ltd.; catalog number: PCRAD1106) contains synthetic nucleic acids for all four targets in a lyophilized format and needs to be reconstituted before use. The negative control (Credo Diagnostics Biomedical Sample Collection Buffer A, Credo Diagnostics Biomedical Pte. Ltd.; catalog number: PCRAE138) consists of the blank transport medium. Both controls are processed according to the same manufacturer’s recommendations as for the patient specimens. A valid positive QC result is demonstrated by the detection of all four viral targets, while a valid negative QC result is confirmed by the detection of only the internal control (IC). Together, these outcomes verify the integrity of the assay reagents and the proper functioning of the system.

### 2.4. Comparative Practicability of VitaSIRO solo™ Instrument, Cepheid GeneXpert^®^ Xpress System, and VitaPCR™ Instrument

**Figure 3:**
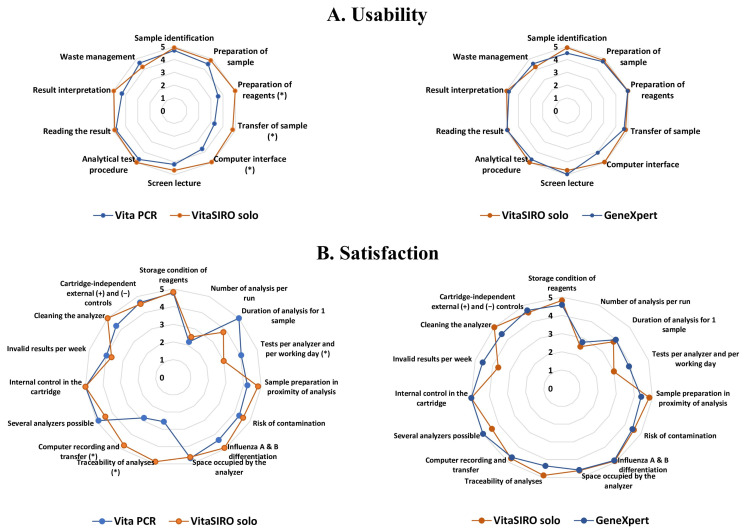
Usability (**A**) and satisfaction questionnaire (**B**) of the VitaSIRO *solo*™ Instrument (Credo Diagnostics Biomedical Pte. Ltd.), the Cepheid GeneXpert^®^ Xpress System (Cepheid Diagnostics), and the first-generation VitaPCR™ Instrument (Credo Diagnostics Biomedical Pte. Ltd.), for the qualitative molecular diagnosis of 4 common respiratory viruses (SARS-CoV-2, influenza A and B viruses, and RSV), among 6 technicians and 4 biologists. The star in brackets indicates significant difference.

The practicability evaluation of the three platforms was divided into two sub-studies carried out by trained health care professionals:

- Substudy 1: Usability evaluation. The usability of each platform was assessed among volunteer health workers from the laboratory, including 6 laboratory technicians and 4 biologists. The participants were first trained in the three platforms, and everyone carefully read the instructions for each kit. The volunteers performed at least 5 measurements with each system, then completed the usability grid comprising 10 items (Figure 3).
- Substudy 2: Satisfaction questionnaire. Afterwards, the participants filled out the satisfaction questionnaire concerning their experiences with each platform, comprising 15 items (Figure 3).

The second-generation VitaSIRO *solo*™ Instrument was compared to the first-generation VitaPCR™ Instrument. Afterwards, the VitaSIRO *solo*™ Instrument was compared to the Cepheid GeneXpert^®^ Xpress System, which was chosen as reference comparator point-of-care analyzer.

Each usability and satisfaction item received a note using an arbitrary quantitative five-point Likert scale [66], ranging from 1 (very difficult), 2 (difficult), 3 (relatively easy), and 4 (easy) to 5 (very easy or comfortable).

### 2.5. Verification of Method According to EN ISO 15189:2022 Criteria for Accreditation

Accreditation of a point-of-care molecular test for multiplex detection of SARS-CoV-2, influenza A virus, influenza B virus, and RSV is governed by the international EN ISO 15189:2022 norma [64,65]. One of the essential steps of accreditation is to carry out on-site method validation and verification. Thus, the laboratory must verify that the VitaSIRO *solo*™ SARS-CoV-2/Flu/RSV Assay performs as intended for each target (SARS-CoV-2, influenza A virus, influenza B virus, and RSV) and works correctly after installation in the laboratory. The on-site verification corresponds to the point 7.3 (“Examination processes”) of the EN ISO 15189:2022 norma [65], and should include the following: (i) Checking for consistent results when tested by the same operator on the same day (repeatability); (ii) checking for consistent results when tested by different operators, on different days, or with different reagent lots (reproducibility); (iii) assessment of the clinical sensitivity to confirm that the test can reliably correctly detect all targets (SARS-CoV-2, influenza A and B viruses, and RSV) using actual patient samples; (iv) assessment of the clinical specificity with patients samples, including insuring that the test does not cross-react with other common respiratory pathogens; and (v) concordance and agreement with gold standard or reference methods.

The repeatability and reproducibility were carried out by repeating 25-fold the positive control supplied by the manufacturer. The resulting coefficients of variation = (standard deviation/mean) × 100 (CV%) were calculated.

### 2.6. Statistical Analysis

Data were entered into an Excel database. Means and standard deviations (SD) were calculated for quantitative variables and proportions for categorical variables. The results were presented along with their two-sided 95% confidence interval (CI) using the modified Wald method for paired data [67]. Contingency tables (2 × 2) were derived for each VitaSIRO *solo*™ target against the comparator reference tests.

Firstly, the mean scores of each item of the usability and satisfaction evaluations, were compared between the VitaSIRO *solo*™ Instrument and VitaPCR™ Instrument, and between the VitaSIRO *solo*™ Instrument and the Cepheid GeneXpert^®^ Xpress System, using the Mann–Whitney U test.

Secondly, the results of SARS-CoV-2, influenza A and B viruses, and RSV detection by the comparator assays were used as the reference standard to estimate the sensitivity and specificity of the VitaSIRO *solo*™ SARS-CoV-2/Flu/RSV Assay, with corresponding 95% CI. The concordance between the VitaSIRO *solo*™ SARS-CoV-2/Flu/RSV Assay and the reference comparator assays was assessed by percent agreement corresponding to the observed proportion of identical results between VitaSIRO *solo*™ SARS-CoV-2/Flu/RSV Assay and the comparator assays. The reliability between the VitaSIRO *solo*™ SARS-CoV-2/Flu/RSV Assay and the comparator assays was estimated by Cohen’s κ coefficient [68], and the degree of agreement was determined as ranked by Landis and Koch [69]. The accuracy of the VitaSIRO *solo*™ SARS-CoV-2/Flu/RSV Assay to correctly diagnose SARS-CoV-2, influenza A and B viruses, and RSV was estimated by Youden’s J index (J = sensitivity + specificity − 1) [70]. Positive predictive value (PPV) and negative predictive value (NPV) were calculated for the VitaSIRO *solo*™ SARS-CoV-2/Flu/RSV Assay by the following formulae [71]: PPV = Number of true positives/Number of true positives + Number of false positives, and NPV = Number of true negatives/Number of true negatives + Number of false negatives.

Thirdly, quantitative agreement and correlations analyses were further assessed. The agreement between the VitaSIRO *solo*™ SARS-CoV-2/Flu/RSV Assay and NeuMoDx™ Flu A-B/RSV/SARS-CoV-2 Vantage Assay was depicted by difference plots as proposed by Bland and Altman [72,73]. The Bland–Altman analyses were carried out to calculate the mean of absolute bias and limits of agreement, respectively, corresponding to the 95% CI [±1.96 × standard deviation (SD)] of the mean absolute bias of all paired measurements [73]. The correlations between the Ct values of target genes obtained by the VitaSIRO *solo*™ SARS-CoV-2/Flu/RSV Assay and the reference NeuMoDx™ Flu A-B/RSV/SARS-CoV-2 Vantage Assay were established by the Passing–Bablok nonparametric linear regression method [74]. The two-side Kendall’s τ test was used to evaluate the level of correlation between the Ct values.

Statistical analyses were performed using Method Validator software version 1.1.9.0. (Philippe Marquis, Metz, France), and online statistical softwares [BiostaTGV (https://biostatgv.sentiweb.fr/, accessed on 7 August 2025) and GraphPad Prism (version 10.6.0; http://www.graphpad.com)]. The *p*-value < 0.05 was considered as statistically significant.

## 3. Results

### 3.1. Repeatability, Reproducibility, and Invalid Results

Repeatability percentages ranged from 2.34% to 4.49%, while reproducibility percentages were between 2.78% and 5.71%.

The VitaSIRO *solo*™ Instrument yielded invalid results in 9 (9/310, 2.9%) specimens, remaining invalid on repeat testing in only 2, which were found negative after 10-fold dilution. All of these specimens were found to be negative with the reference NeuMoDx™ assay. We have kept the final results of these samples in the subsequent analyses.

### 3.2. Comparative Usability and Satisfaction Evaluation

The results of usability and satisfaction of the VitaSIRO *solo*™ Instrument, the first generation VitaPCR™ Instrument, and the Cepheid GeneXpert^®^ Xpress System are shown in Figure 3.

The usability results were comparable for VitaSIRO *solo*™ Instrument and Cepheid GeneXpert^®^ Xpress System, with slight differences, which, however, did not reach statistical significance. In contrast, the preparation of sample, transfer of the sample to the reagent tube or cartridge, and the intuitiveness of the computer interface were considered slightly less easy for the VitaPCR™ Instrument than the VitaSIRO *solo*™ Instrument (item “sample preparation”: 3.6 ± 1.1 versus 5.0 ± 0.0, *p* < 0.05; item “sample transfer to reagent tube or cartridge”: 3.3 ± 0.5 versus 4.7 ± 0.3; *p* < 0.02; item “computer interface”: 3.6 ± 0.5 versus 4.9 ± 0.1, *p* < 0.002).

The satisfaction questionnaire showed comparable results for VitaSIRO *solo*™ Instrument and Cepheid GeneXpert^®^ Xpress System, with slight and not significant differences. In contrast, the score for the item “number of tests per analyzer and per working day” was higher for the VitaPCR™ Instrument than for the VitaSIRO *solo*™ Instrument (4.1 ± 0.3 versus 3.0 ± 0.0, *p* < 0.001). Indeed, the short duration of analysis for one sample was the most appreciated item, with only 20 min for the VitaPCR™ Instrument against 40 min for the VitaSIRO *solo*™ Instrument. The computer system appeared to be clearly less efficient for the VitaPCR™ Instrument than for the VitaSIRO *solo*™ Instrument (item “traceability of analyses”: 2.5 ± 1.3 versus 4.9 ± 0.3, *p* < 0.001; item “computer recording and transfer”: 2.8 ± 1.2 versus 4.8 ± 0.3, *p* < 0.001). The other items of satisfaction gave comparable scores for the VitaPCR™ Instrument and the VitaSIRO *solo*™ Instrument.

### 3.3. Analytical Performances Using Clinical Samples

#### 3.3.1. Results by Reference Assays

According to the final results of the reference multiplex molecular assays, from a total of 301 respiratory samples collected during the 2024–2025 autumn–winter season, 175 (58.2%) samples were positive for at least one respiratory virus including 39 (12.9%) samples for SARS-CoV-2, 97 (32.2%) for influenza viruses, with 77 (25.5%) for influenza A virus and 20 (6.7%) for influenza B virus, and 42 (13.9%) for RSV, whereas 126 samples (41.8%) were negative for SARS-CoV-2, influenza A and B viruses, and RSV. The majority of samples were single-infected, while three (0.9%) samples positive for SARS-CoV-2 were co-infected by influenza A virus (*n* = 1; W3 of 2025), influenza B virus (*n* = 1; W5 of 2025), and RSV (*n* = 1; W49 of 2024). SARS-CoV-2 infection was present throughout the inclusion period, without epidemic variation, but with slight peaks at the end of summer (W39) and during the winter (W51 of 2024 to W6 of 2025). Influenza infection was clearly epidemic, extending over a 15-week period, from W47 of 2024 to W10 of 2025. Remarkably, the curve describing the influenza epidemic appeared biphasic, with a first peak in January 2025 and a second peak in February 2025. A total of 45% (9 out of 20) of influenza B infections were detected during the second influenza peak, in February 2025, with the remaining cases distributed from November 2024 to the end of January 2025. RSV infection was also clearly epidemic, from week W46 in November 2024 to week W3 in January 2025, with a peak in week W48; this epidemic preceded the influenza epidemic by a fortnight.

#### 3.3.2. Analytical Performances of the VitaSIRO *solo*™ SARS-CoV-2/Flu/RSV Assay (Table 1)

Out of 39 respiratory specimens positive for SARS-CoV-2 by reference assays, 37 were detected by the VitaSIRO *solo*™ SARS-CoV-2/Flu/RSV Assay (targeting the N gene), with no false positives. The two false negatives had Ct values > 33 (Ct 34.6 and 35.0).

For influenza, 77 and 20 specimens tested positive for influenza A and B, respectively, by reference methods. The VitaSIRO *solo*™ assay detected 75 influenza A (M gene) and 18 influenza B (NS gene) cases, resulting in four false negatives. Three of these had Ct > 33 (33.7, 33.8, and 35.0), while one had Ct 31.8. One influenza A false positive was observed (Ct 34.0).

Among 42 RSV-positive specimens by reference assays, 40 were detected by the VitaSIRO *solo*™ assay (N gene), with no false positives. The two false negatives had Ct values of 33.7 and 34.1.

Among the 175 virus-positive specimens, 8 (4.6%) were false negatives; among 126 virus-negative specimens, 1 (0.8%) was a false positive. The overall sensitivities and specificities of the VitaSIRO *solo*™ SARS-CoV-2/Flu/RSV Assay were 94.8% and 100.0%, respectively, for SARS-CoV-2 (with Ct ≤ 33), 95.8% and 99.5%, respectively, for influenza A and B viruses, 95.2% and 100.0%, respectively, for RSV, and 95.4% and 99.2%, respectively, for all tested respiratory viruses.

Among the 125 samples negative for SARS-CoV-2, influenza A and B viruses, and RSV by Vi-taSIRO *solo*™ SARS-CoV-2/Flu/RSV Assay and reference assays, a total of 8 specimens of 28 (28.5%), which were also tested by FilmArray Respiratory Panel 2.1 on request from clinicians, were positive for other respiratory pathogens, including 5 for rhinovirus, 2 for adenovirus, and 1 for parainfluenza 3 virus. None were found positive for SARS-CoV-2, influenza A and B viruses, or RSV.

**Table 1:** Analytical performances of the VitaSIRO *solo*™ SARS-CoV-2/Flu/RSV assay (Credo Diagnostics Biomedical Pte. Ltd.) by reference to the results from all the reference comparator assays, including the NeuMoDx™ Flu A-B/RSV/SARS-CoV-2 Vantage Assay (Qiagen GmbH), the FilmArray Respiratory Panel 2.1 (Bio-Fire Diagnostics, Salt Lake City, UT, USA), and the Xpert^®^ Xpress CoV-2 plus test (Cepheid Diagnostics).

				VitaSIRO *solo*™ SARS-CoV-2/Flu/RSV Assay									
				*n*	Positive (*n*)	Negative (*n*)	Sensitivity (% [95%CI]) ^µ^	Specificity (% [95%CI])	Agreement ^a^ (% [95%CI])	Concordance ^b^ (% [95%CI])	Youden’ J Index ^c^ (% [95%CI])	PPV ^d^ (% [95%CI])	NPV ^d^ (% [95%CI])
Reference assays	SARS-CoV-2	Positive	All Ct ^$^ values	39	37	2	94.8 [82.2–99.4]	100.0 [98.2–100.0]	99.3 [97.4–99.9]	0.970 [0.928–1.0]	0.948 [0.822–0.994]	100.0 [88.8–100.0]	99.2 [97.1–99.9]
			≤33	34	34	0	100.0 [87.9–100.0]	100.0 [98.2–100.0]	100.0 [99.8–100.0]	1.0 [0.999–1.0]	1.0 [0.982–1.0]	100.0 [87.9–100.0]	100.0 [98.2–100.0]
			>33	5	3	2	60.0 [22.9–88.4]	100.0 [98.2–100.0]	99.2 [97.1–99.9]	0.746 [0.408–1.0]	0.600 [0.229–0.884]	100.0 [38.2–100.0]	99.2 [97.1–99.9]
		Negative		262	0	262	-	-	-	-	-	-	-
	Influenza A	Positive		77	75	2	97.4 [90.4–99.8]	99.5 [97.2–99.9]	99.0 [96.9–99.8]	0.974 [0.944–1.0]	0.969 [0.931–0.989]	98.6 [92.2–99.9]	99.1 [96.6–99.9]
		Negative		224	1	223							
	Influenza B	Positive		20	18	2	90.0 [68.6–98.4]	100.0 [98.6–100.0]	99.3 [97.4–>99.9]	0.944 [0.866–1.0]	0.900 [0.686–0.984]	100.0 [79.3–100.0]	99.2 [97.2–99.9]
		Negative		281	0	281							
	Influenza A/B	Positive		97	93	4	95.8 [89.5–98.7]	99.5 [98.6–100.0]	98.3 [96.0–99.74	0.962 [0.928–0.995]	0.953 [0.914–0.977]	98.9 [93.6–99.9]	98.0 [94.9–99.4]
		Negative		204	1	203							
	RSV	Positive		42	40	2	95.2 [83.3–99.5]	100.0 [99.9–100.0]	99.3 [97.84–>99.9]	0.972 [0.933–1.0]	0.952 [0.833–0.995]	100.0 [89.5–100.0]	99.2 [97.0–99.9]
		Negative		259	0	259							
	SARS-CoV2 + influenza A/B + RSV	Positive		175	167	8	95.4 [91.1–97.8]	99.2 [99.7–100.0]	97.0 [94.3–98.5]	0.939 [0.900–0.978]	0.946 [0.906–0.969]	99.4 [94.5–99.9]	93.9 [88.4–97.0]
		Negative		126	1	125							

^a^ Agreement = TP + TN/TP + FP + TN + FN, expressed in percentage; ^b^ The Cohen’s κ coefficient calculation was used to estimate the concordance [68] and interpreted according the Landis and Koch scale [69], as follows: <0 as indicating no agreement, 0–0.20 as slight, 0.21–0.40 as fair, 0.41–0.60 as moderate, 0.61–0.80 as substantial, and 0.81–1 as almost perfect concordance; ^c^ The accuracy of the VitaSIRO *solo*™ platform to correctly diagnose respiratory virus infections was estimated by Youden’s J index (J = sensitivity + specificity − 1) [70]; ^d^ PPV = TP/TP + FP and NPV = TN/TN + FN; ^µ^ 95% confidence intervals in brackets were calculated by using the modified Wald method for paired data [67]; ^$^ The Ct values of Nsp2 gene detection by the NeuMoDx™ Flu A-B/RSV/SARS-CoV-2 Vantage Assay (Qiagen GmbH, Hilden, Germany) were used to classify nasopharyngeal samples according to their level of SARS-CoV-2 RNA excretion; Ct < 33 a.u. was taken as threshold of high SARS-CoV-2 RNA excretion, as previously stated [75,76]. a.u.: Arbitrary unit; CI: Confidence interval; Ct: Cycle threshold; NPV: Negative predictive value; PPV: Positive predictive value; and RSV: Respiratory syncytial virus.

### 3.4. Agreement Between VitaSIRO solo™ SARS-CoV-2/Flu/RSV Assay and Comparator NeuMoDx™ Flu A-B/RSV/SARS-CoV-2 Vantage Assay

For SARS-CoV-2 infection, the VitaSIRO *solo*™ SARS-CoV-2/Flu/RSV Assay showed almost perfect agreement (99.3%), high reliability assessed by Cohen’s κ coefficient (0.970), and excellent accuracy assessed by Youden’s J index (0.948) to detect SARS-CoV-2 RNA as well as high PPV and NPV of 100.0% and 99.2%, respectively. These analytical performances were further stratified according to the Ct values of the Nsp2 gene detected by the reference NeuMoDx™ assay, considering Ct-related criteria of high (Ct ≤ 33 a.u.) SARS-CoV-2 RNA excretion [75,76]. In the event of high viral excretion, the analytical performances remained stable and excellent. In the event of moderate or very low viral excretion (Ct > 33 a.u.), the sensitivity, concordance, and accuracy dropped slightly, while the specificity, PPV, and NPV remained constant.

For influenza A and B infections as well as RSV infection, the VitaSIRO *solo*™ SARS-CoV-2/Flu/RSV Assay showed similarly high PPV, NPV, agreement, reliability, and accuracy, with an overall high agreement for influenza A and B viruses, and RSV, and high Cohen’s κ coefficient ranging from 0.939 to 0.974, indicating an almost perfect agreement between assays.

### 3.5. Association of Ct Values Between VitaSIRO solo™ SARS-CoV-2/Flu/RSV Assay and Comparator NeuMoDx™ Flu A-B/RSV/SARS-CoV-2 Vantage Assay

**Figure 4:**
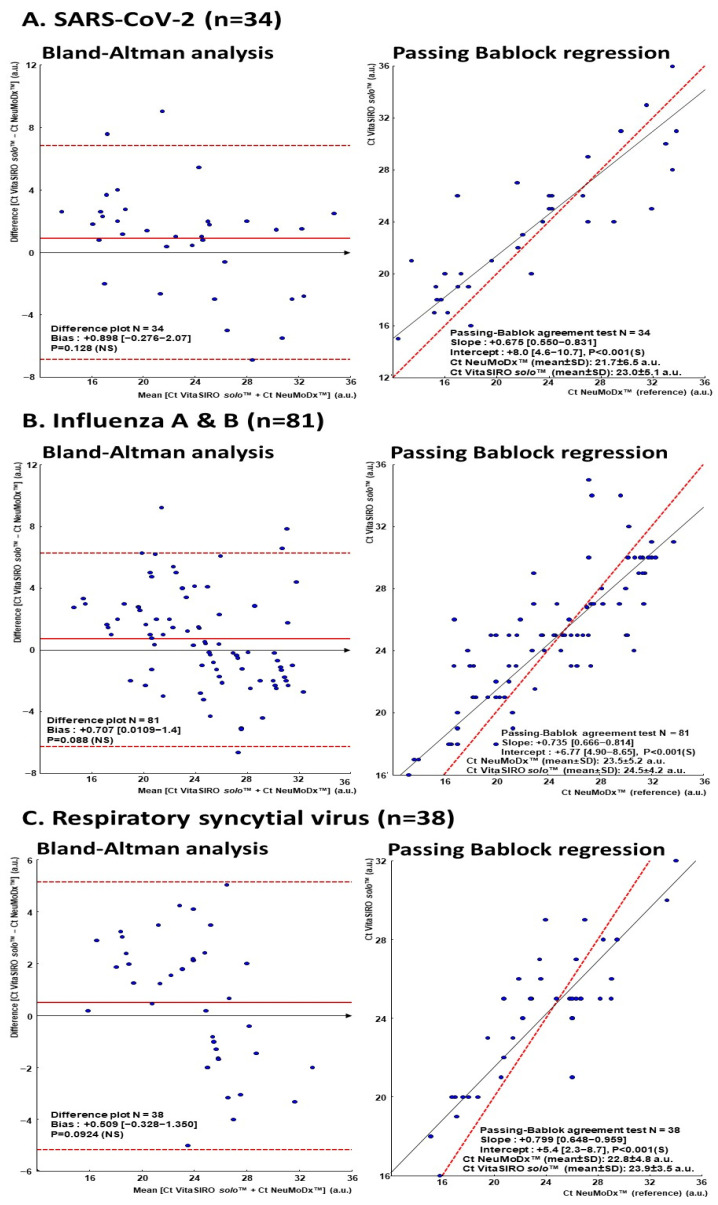
Bland–Altman analyses on the relative differences between the Ct values obtained by the VitaSIRO *solo*™ SARS-CoV-2/Flu/RSV Assay (Credo Diagnostics Biomedical Pte. Ltd.) and the NeuMoDx™ Flu A-B/RSV/SARS-CoV-2 Vantage Assay (Qiagen GmbH), used as reference comparator assay, and Passing–Bablok nonparametric linear regression curves between the Ct values obtained by the VitaSIRO *solo*™ SARS-CoV-2/Flu/RSV Assay and the reference NeuMoDx™ Flu A-B/RSV/SARS-CoV-2 Vantage Assay, calculated from Ct value results of paired detection of SARS-CoV-2 (**A**), influenza A and B viruses (**B**), and RSV (**C**), in archived predetermined clinical respiratory samples. For Passing–Bablok nonparametric linear regression curves, the diagonal dotted line represents the ideal line (no bias), whereas the full line represents the regression line of the distribution. For the Bland–Altman analyses, the full line represents the mean relative difference, the dotted lines represent the superior and inferior limits of agreement, and the arrow corresponds to the x abscise axis. Quantitative results are Ct values in arbitrary units (a.u.) for each virus or target gene detected. Only results from samples positive by both VitaSIRO *solo*™ SARS-CoV-2/Flu/RSV Assay Credo and reference NeuMoDx™ Flu A-B/RSV/SARS-CoV-2 Vantage Assay were used for analyses. Ct: Cycle threshold; RSV: Respiratory syncytial virus.

Mean absolute bias and their limits of agreement were measured by Bland–Altman analyses between Ct values of samples, that tested positive by VitaSIRO *solo*™ SARS-CoV-2/Flu/RSV Assay and reference NeuMoDx™ assay, are depicted in Figure 4 for SARS-CoV-2, influenza A and B viruses, and RSV, from the Ct values of both assays (Table 2). A total of 91.1% (31/34), 94.8% (73/77), and 100.0% (38/38) of values were within the 95% limits of agreement for the detection of SARS-CoV-2, influenza A and B viruses, and RSV, respectively, demonstrating a good agreement between VitaSIRO *solo*™ and NeuMoDx™ assays. The mean absolute bias ranged from +0.509 for RSV and +0.707 for influenza A and B viruses to +0.898 for SARS-CoV-2, showing a slight increase in Ct with the VitaSIRO *solo*™ SARS-CoV-2/Flu/RSV Assay, however, without significant statistical difference with the comparator assay.

The non-parametric Passing–Bablok regression analyses between the Ct values of samples positive for SARS-CoV-2, influenza A and B viruses, or RSV by VitaSIRO *solo*™ SARS-CoV-2/Flu/RSV Assay and the NeuMoDx™ comparator assay are shown in Figure 4. The Ct values of positive samples by the two assays were significantly correlated for SARS-CoV-2 (Kendall’ τ = 0.672; *p* < 0.001), influenza A and B viruses (τ = 0.647; *p* < 0.001), and RSV (τ = 0.491; *p* < 0.001). The slopes ranged from +0.675 for SARS-CoV-2 and +0.700 for RSV to +0.735 for influenza A and B viruses, and their intercepts ranged from +5.4 for RSV and +6.7 for influenza A and B viruses to +8.0 for SARS-CoV-2.

As a whole, although the Ct values obtained were positively correlated and the small positive bias in favor of VitaSIRO *solo*™ SARS-CoV-2/Flu/RSV Assay was not significant, the VitaSIRO *solo*™ assay tended to overestimate low Ct values by the reference NeuMoDx™ assay, while it tended to underestimate high Ct values.

## 4. Discussion

With a view to accreditation according to the analytical criteria of the 2022-revised EN ISO 15189:2022 norma, with the aim of improving the quality of care, we herein retrospectively assessed the practicability, the on-site repeatability and reproducibility, and the clinical analytical performances, as well as the concordance agreement with reference molecular assays, of the multiplex rRT-PCR VitaSIRO *solo*™ SARS-CoV-2/Flu/RSV Assay performed on the VitaSIRO *solo*™ Instrument for the simultaneous detection of SARS-CoV-2, influenza A and B viruses, and RSV in respiratory specimens collected during the 2024–2025 autumn–winter season in Paris, France. The temporal variation in detected viruses over a 35-week period showed sustained presence of SARS-CoV-2 with slight peaks, contrasting with the distinct epidemic patterns observed for of the RSV epidemic with earlier onset and end compared to the epidemics of influenza A and B viruses, qualifying as so-called “tripledemics” with concurrent outbreaks of SARS-CoV-2, influenza viruses, and RSV. Comparative usability and satisfaction evaluations highlighted the competitive nature of the VitaSIRO *solo*™ Instrument against established point-of-care platforms. The observed repeatability and reproducibility percentages for all target genes demonstrated a high level of precision for the VitaSIRO *solo*™ platform, consistent with the stringent requirements for clinical diagnostic assays [77,78]. The VitaSIRO *solo*™ SARS-CoV-2/Flu/RSV Assay showed high level of sensitivity, specificity, agreement, reliability, and accuracy with reference comparator assays for the qualitative detection and differentiation of SARS-CoV-2, influenza A and B viruses, and RSV across a wide range of tested Ct values of the four viruses. Furthermore, significant positive correlations of Ct values were observed for positive samples of the four viruses between the VitaSIRO *solo*™ SARS-CoV-2/Flu/RSV Assay and the reference comparator NeuMoDx™ assay, with an overall high agreement between Ct values assessed by Bland–Altman analyses. Altogether, these findings demonstrate that the multiplex VitaSIRO *solo*™ SARS-CoV-2/Flu/RSV Assay performed on the VitaSIRO *solo*™ Instrument constitutes a reliable test to detect and differentiate simultaneously SARS-CoV-2, influenza A and B viruses, and RSV in respiratory specimens, featuring analytical and clinical performances comparable to currently used molecular methods for the detection of these pathogens in clinical specimens. The ability to screen for all four viruses in a single reaction allows for streamlined workflows and conservation of resources during influenza-seasons. Taken together, the novel second-generation point-of-care VitaSIRO *solo*™ Instrument demonstrates favorable usability, high analytical precision, and robust performance, positioning it as a valuable tool for rapid and accurate multiplex detection of four common respiratory viruses. Its high concordance with reference molecular tests and the epidemiological trends observed in tracking epidemic variations in the four main respiratory viruses, as in the autumn–winter of 2024–2025 in France, underline its potential for effective clinical diagnosis, including in decentralized environments, and for public health surveillance.

1°. Practicability. The practicability of the VitaSIRO *solo*™ Instrument, the first generation VitaPCR™ Instrument, and the Cepheid GeneXpert^®^ Xpress System was assessed by usability and satisfaction questionnaires completed by trained health care professionals. The platform Cepheid GeneXpert^®^ Xpress System was used as a reference comparator point-of-care analyzer [63]. On the whole, both VitaSIRO *solo*™ SARS-CoV-2/Flu/RSV Assay and Xpert^®^ Xpress CoV-2 plus test, with their dedicated platforms, were easy to use and received excellent appreciation for practical routine use. All reagents were pre-filled in the cartridge. Both assays required only, approximately, around 2 to 5 min of hands-on time. The transfer of the sample to the reagent tube and the intuitiveness of the computer interface were considered similarly easy for the VitaSIRO *solo*™ Instrument than the Cepheid GeneXpert^®^ Xpress System. The differentiation between SARS-CoV-2, influenza A and B viruses, and RSV was unambiguous for both platforms, with the possibility to diagnose co-infection. The closed conception of both VitaSIRO *solo*™ Instrument and Cepheid GeneXpert^®^ Xpress System helped mitigate the cross-contamination issues that can be problematic with other molecular-analysis-based assays. Both platforms deliver results in a relatively similar time (VitaSIRO *solo*™: 40 min; Cepheid Xpert^®^: 36 min). Finally, our findings confirm high usability and excellent appreciation of both the VitaSIRO *solo*™ Instrument and Cepheid GeneXpert^®^ Xpress System, with the little place occupied, and the possibility of point-of-care use. The two limiting factors were the relatively long analysis time (over 30 min), which is the principal criterion for classifying a test as “rapid” [79,80], and the fact that each analysis was performed on a per-unit basis. However, both platforms are designed to integrate several modules together, making it easy to multiply analyses. Finally, the comparative usability and satisfaction evaluations highlighted the competitive nature of the VitaSIRO *solo*™ Instrument against established platforms. The comparable usability results between the VitaSIRO *solo*™ Instrument and Cepheid GeneXpert^®^ Xpress System suggest that the novel system maintains a user-friendly interface and streamlined workflow, which are critical for adoption in diverse laboratory settings [81]. Otherwise, the comparison of the VitaSIRO *solo*™ Instrument with the first generation VitaPCR™ Instrument allowed evaluating the technological advances in the design of these two platforms. While the first-generation VitaPCR™ Instrument showed some limitations in sample preparation, transfer of the sample to the reagent tube, and computer interface intuitiveness, the VitaSIRO *solo*™ Instrument addressed these aspects effectively, signifying valuable design improvements. The higher satisfaction score for the VitaPCR™ Instrument regarding the “number of tests per analyzer and per working day” and its shorter analysis time per sample (20 min versus 40 min for VitaSIRO *solo*™ Instrument) suggests that throughput remains an important consideration for high-volume testing scenarios. However, the VitaSIRO *solo*™ Instrument demonstrated superior performance in terms of traceability and computer recording/transfer, indicating advancements in data management and quality control features, which are increasingly vital in modern molecular diagnostics [82,83]. Taken together, the format of the second-generation VitaSIRO *solo*™ Instrument and its ease of use make its integration into various laboratory facilities reasonably simple, from routine clinical laboratories to urgent laboratories (with results within 1–4 h), in areas such as emergency departments [84,85] or congregate living settings [86], and even in rapid-response STAT laboratories (with results within 1 h or less), where the assay can easily be performed upon receipt of the specimen (on demand), with results available in less than 1 h. The overwhelmingly positive usability feedback likely supports the potential for important adoption in various decentralized diagnosis testing settings, such as hospital and emergency room, nursing home, clinic, airport, factory, cruise, warships, or oil platforms. External quality control tests supplied by the manufacturers allow, at a frequency chosen by each laboratory, integrated quality control assurance to manage any risk of error during the analysis, which is required by the EN ISO 15189:2022 norma [65,82,83,87]. Finally, the VitaSIRO *solo*™ Instrument may also be deployed in facilities where licensed technologists may not be available or where technologists are unfamiliar with high-complexity PCR assays, especially in resource-limited settings, as an example.

2°. Repeatability, reproducibility, and invalid results. The observed repeatability and reproducibility percentages for all target genes (ranging from 2.34% to 4.49% and 2.78% to 5.71%, respectively) demonstrate a high level of precision for the VitaSIRO *solo*™ Instrument, which is consistent with the stringent requirements for clinical diagnostic assays [77]. Indeed, a repeatability of less than 5% is considered excellent for intra-assay measurements in rRT-PCR [77,78]. Reproducibility, in real-time RT-PCR, refers to the precision of results obtained from identical samples tested under different conditions (e.g., different operators, different instruments, different days, or even different laboratories). It is a broader measure of precision than repeatability, and it is crucial for demonstrating the robustness and transferability of an assay. For inter-assay (within-laboratory, but different runs/days/operators) reproducibility, a CV% of less than 10%, as for the VitaSIRO *solo*™ SARS-CoV-2/Flu/RSV Assay performed on the VitaSIRO *solo*™ Instrument, is generally considered acceptable and often targeted for rRT-PCR assays, especially for validated diagnostic kits or methods [78,88].

The low incidence of invalid results (2.9%), with only a minor proportion remaining invalid on repeat testing and subsequently confirmed negative by a reference assay, indicates robust assay performance and minimal sample-related interference. This invalid results rate was two-fold lower than that reported for the VitaPCR™ Instrument (5.9%) [63]. The extraction step followed by purification step with the VitaSIRO *solo*™ Instrument likely eliminates the majority of PCR inhibitors from the mucosal secretions, thus reducing the risk of invalid results. This low rate of invalidation is crucial for maintaining efficient laboratory workflows and minimizing the need for retesting, which can contribute to faster turnaround times for patient results [89]. High-performing point-of-care molecular assays should ideally have a very low invalid rate, often around 5–6% [90], and, ideally, closer to 1–2% or less [91], with efforts to resolve initial invalid results through retesting where appropriate. Any higher sustained rate of invalid results would trigger an investigation into potential issues with the device, reagents, or user training and compliance.

3°. Analytical performance. The analytical performance of the VitaSIRO *solo*™ SARS-CoV-2/Flu/RSV Assay using clinical samples demonstrated high sensitivity and specificity. The overall sensitivities of 94.8% for SARS-CoV-2 (Ct ≤ 33 a.u.), 95.8% for influenza A and B viruses, 95.2% for RSV, and 95.4% for all tested respiratory viruses, coupled with specificities ranging from 99.2% to 100.0%, underscore the assay’s clinical utility for accurate respiratory virus detection. The high concordance between the VitaSIRO *solo*™ assay and the reference NeuMoDx™ assay, evidenced by almost perfect agreement and high Cohen’s κ coefficients for all targets (ranging from 0.939 to 0.974), further substantiates its reliability. Taken together, the overall performance of the VitaSIRO *solo*™ SARS-CoV-2/Flu/RSV Assay was highly comparable to that of the reference multiplex rRT-PCR assays for the qualitative detection and differentiation of SARS-CoV-2, influenza A and B viruses, and RSV in respiratory specimens.

There were only nine discordant samples for SARS-CoV-2, influenza A and B viruses, and RSV. The risk of false-negative results is one of the major issues of the diagnosis of respiratory viral infections, as, for example, when using currently available rapid antigenic tests for influenza [92]. The majority of false negative discrepant samples showed seemingly high Ct values by reference to the comparator NeuMoDx™ assay, suggesting that the samples had a low viral load, providing analytical limitations of nucleic acid amplification tests at the lower limits of detection [93], or that the negative testing result could be due to multiple freeze–thaw steps of sample processing, which has a significant impact on molecular detection [94]. Furthermore, negative sample results cannot exclude inadequate sampling, or inadequate sample integrity of targets [95]. The introduction of encapsulated RNA as internal control in the VitaSIRO *solo*™ SARS-CoV-2/Flu/RSV Assay allowed this, however, confirming, in part, adequate sample specimens and appropriate testing conditions. In our hands, the PPV of VitaSIRO *solo*™ SARS-CoV-2/Flu/RSV Assay for influenza A or B virus detection was near 100%, demonstrating that a positive test constitutes a true case of flu during the seasonal influenza epidemic. Otherwise, the NPVs of VitaSIRO *solo*™ SARS-CoV-2/Flu/RSV Assay for influenza A and B viruses were around the threshold of 98%, indicating that a negative test result indicates a high probability that the individual is indeed healthy from flu. The possibility of false negative results always exists, especially if the infectious viral load is low, or, also, because of the existence of a new emerging variants harboring mutations responsible for genetic variation which could compromise the diagnostic performance of a molecular test. If a negative test does not completely rule out the diagnosis of influenza during the epidemic season, the clinician should repeat the molecular test or request the use of another molecular test if influenza-like clinical conditions persist, and he should also look for other respiratory viruses, including SARS-CoV-2 or RSV. All in all, maximizing confidence in a negative result is an important issue of public health, especially in the cases where false-negative results are reported for pathogens having a high potential of infection spread [52].

4°. Agreement and correlation. Bland–Altman and Passing–Bablok regression analyses revealed a good agreement and significant correlation between the Ct values obtained by the VitaSIRO *solo*™ and NeuMoDx™ assays. As more than 95% of values were within the 95% limits of agreement, both assays can be used interchangeably for concurrent detection of SARS-CoV-2, influenza A and B viruses, and VRS. While a slight positive bias was observed for the VitaSIRO *solo*™ assay, indicating a tendency to yield slightly higher Ct values, this difference did not reach statistical significance. The observation that the VitaSIRO *solo*™ assay tends to overestimate low Ct values and underestimate high Ct values, while not statistically significant in this analysis, warrants consideration in clinical interpretation, particularly when the estimation of viral load quantification using Ct values as a proxy of genomic load is critical to evaluate the viral shedding [96,97]. However, the correlation observed across the range of Ct values suggests that the VitaSIRO *solo*™ assay provides consistent semi-quantitative results comparable to a well-established reference method.

5°. Accreditation criteria. The EN ISO 15189:2022 norma emphasizes the need for laboratories to validate examination procedures and establish the performance characteristics of their methods, including precision [repeatability (intra-assay variation) and reproducibility (inter-assay variation or variation under different conditions)] and the handling of invalid results [65]. Both the repeatability and reproducibility percentages and the analysis and handling of invalid results, with a low rate of persistent invalid results (2 out of 310), directly corresponded with the criteria for accreditation according to the EN ISO 15189:2022 norma. These aspects demonstrate that the laboratory has characterized the precision level of the on-site use of the VitaSIRO *solo*™ Instrument, and has a system for managing non-conforming results, which are fundamental requirements for accreditation. Otherwise, the EN ISO 15189:2022 norma requires that methods be validated using clinical samples to confirm performance characteristics in a real-world setting. The use of a reference method (multiplex molecular assays) to define the true positive/negative status of the samples was a key part of this validation process. The determination and reporting of key performance characteristics, specifically sensitivity and specificity based on a clinical sample set with defined reference methods, and the detailed characterization of false negative and false positive results, were all fundamental requirements for accreditation under the EN ISO 15189:2022 norma. The analysis of performance stratified according to the Ct values (high vs. moderate/low viral excretion) demonstrates a thorough understanding of the assay’s performance across different viral loads, which can inform the clinical interpretation of results, especially for samples with low viral concentrations. Thus, the observation that most false negatives occurred with high Ct values (low viral load) helped to define the effective analytical performance of the assay, and to understand the limitations of an accredited test. Finally, the detailed comparison of the VitaSIRO *solo*™ Assay with comparator reference methods, along with the comprehensive reporting of agreement, reliability (Cohen’s κ), accuracy (Youden’s J), and predictive values (PPV and NPV), all directly corresponded to the criteria for accreditation under EN ISO 15189:2022 and confirmed the suitability of the VitaSIRO *solo*™ assay for intended use.

6°. Temporal distribution. The temporal distribution of detected viruses during the 2024–2025 autumn–winter season provides remarkable valuable epidemiological insights. The sustained presence of SARS-CoV-2 throughout the inclusion period, with slight peaks, contrasted with the distinct epidemic patterns observed for influenza viruses and RSV. The biphasic influenza epidemic, particularly the later surge of influenza B cases, highlights the dynamic nature of seasonal respiratory virus circulation and the importance of continuous surveillance. First of all, a substantial proportion (58.2%) of the 301 archived respiratory samples were positive for at least one respiratory virus during the study period, underscoring the significant burden of respiratory infections during the 2024–2025 autumn–winter season in hospitalized adult patients suffering from influenza-like signs and symptoms in Paris, with periods of co-circulation of all three major respiratory viruses. Secondly, the temporal occurrence data showed distinct but overlapping epidemic curves. SARS-CoV-2 infection was continuously present throughout the inclusion period, with slight peaks at the end of summer (W39) and during winter (W51 of 2024 to W6 of 2025), suggesting an endemic pattern with seasonal exacerbations. This contrasts with the more pronounced epidemic waves of influenza viruses and RSV. Thus, for RSV infection, the distinct epidemic from W46 in November 2024 to W3 in January 2025, peaking in W48 and preceding the flu epidemic by a fortnight, aligns with typical RSV seasonality, often occurring earlier than influenza in France [98]. For influenza infection, the clear epidemic extending over a 15-week period (W47 of 2024 to W10 of 2025) with a biphasic peak (January and February 2025), highlighting its typical seasonal surge, was potentially influenced by reduced immunity from prior seasons or specific viral dynamics [99]. The second peak with a high proportion of influenza B cases is a notable detail. Our observations at the level of a single hospital in Paris are comparable to official French government epidemiological data collected during the last autumn–winter season in France [98].

The staggered but ultimately overlapping nature of these epidemic curves demonstrate the co-circulation of SARS-CoV-2, influenza A and B viruses, and RSV during the last autumn–winter season in Paris, and the possibility of co-infection, mainly from December to February. Thus, the “post-COVID era” during the 2024–2025 autumn–winter season in France was marked by a complex interplay of respiratory viruses, often leading to what has been termed a “tripledemic” season: the simultaneous circulation and co-infections of SARS-CoV-2, influenza A and B, and RSV [15,16,22]. While not all viruses peak simultaneously, their prolonged co-circulation ensured a continuous high demand on health care resources [22,98].

7°. Study limitations. A limitation of the study includes the relatively small sample size for some viruses (influenza B virus and RSV), with the inability to control for sampling variability. The small number of positive samples for influenza B virus and RSV corresponds, in fact, to the limited epidemics of both viruses during the last 2024–2025 autumn–winter season in France. Second, this was a single-center study, and the VitaSIRO *solo*™ SARS-CoV-2/Flu/RSV Assay needs to be further evaluated in other testing sites. Third, the number of specimens negative for SARS-CoV-2, influenza A and B viruses, and RSV, but positive for other respiratory viruses was low, which makes it difficult to identify any cross-reactivity. Fourth, the study was retrospective and was conducted on frozen samples, which can lead to selection and sample quality bias, because respiratory secretions have been diluted in virus transport medium and because the freeze–thaw step of sample processing may impact molecular detection [94]. Finally, an additional comparator method to discern the discrepancies between VitaSIRO *solo*™ SARS-CoV-2/Flu/RSV Assay and reference multiplex rRT-PCR results was lacking.

## 5. Conclusions

Unprecedented development has been seen in the fields of molecular tools for diagnostics of infectious diseases over the last 5 years, fueled, in part, by the COVID-19 pandemic. Not only were laboratory-based assays to detect SARS-CoV-2 RNA rapidly developed, but several point-of-care platforms became available within less than 2 years of identifying the virus [48,58,61,100], including fully integrated microfluidic devices [45,53,54,101]. Efforts were also facilitated by streamlined regulatory review processes which allowed the tests to be quickly marketed and distributed globally. In the context of somewhat declining demand for COVID-19 testing, developers have been adapting their technologies to address the need to diagnose other infectious diseases with point-of-care systems, especially in the field of respiratory virus infections, thus supporting the expectations of access to immediate diagnostic answers for rapid clinical decision-making, managing appropriate therapy and supporting antimicrobial stewardship by specifically identifying one of many potential causes of infection. In this context of rapidly evolving technology, the VitaSIRO *solo*™ Instrument demonstrates favorable usability, high analytical precision, and robust performance, positioning this point-of-care analyzer as a valuable tool for rapid and accurate multiplex detection of common respiratory viruses responding to the EN ISO 15189:2022 criteria for accreditation. Its strong agreement with comparator reference molecular assays underscores its potential for effective clinical diagnostics in various clinical settings and public health surveillance.

## Figures and Tables

**Table 2:** Cycle threshold (Ct) values of respiratory specimens positive for SARS-CoV-2, influenza A and B viruses, or respiratory syncytial virus (RSV) by the VitaSIRO *solo*™ SARS-CoV-2/Flu/RSV assay and the NeuMoDx™ Flu A-B/RSV/SARS-CoV-2 Vantage Assay.

	SARS-CoV-2 (*n* = 34)	Influenza A and B (*n* = 81)	RSV (*n* = 38)
VitaSIRO *solo*™ SARS-CoV-2/Flu/RSV assay	23.01 ± 5.17 (15 ^µ^–36) ^µµ^	24.55 ± 4.21 (16–35)	23.90 ± 3.49 (16–32)
NeuMoDx™ Flu A-B/RSV/SARS-CoV-2 Vantage Assay	21.74 ± 6.55 (12.4–33.8)	23.59 ± 5.29 (13.2–33.7)	22.88 ± 4.85 (15.1–34.0)
P ^µµµ^	0.128	0.088	0.092

^µ^ Ct values by the VitaSIRO *solo*™ Instrument are whole numbers; ^µµ^ Results as expressed as mean ± SD a.u. (arbitrary unit); range in brackets; ^µµµ^ Difference between mean Ct values by VitaSIRO *solo*™ SARS-CoV-2/Flu/RSV assay and NeuMoDx™ Flu A-B/RSV/SARS-CoV-2 Vantage Assay, using the Wilcoxon signed rank test for paired samples.

## Data Availability

The data that support the conclusions of this study are available from the corresponding author upon reasonable request due to legal limitation of transmitting Excel database.
